# Effect of the Symbolic Meaning of Speed on the Perceived Duration of Children and Adults

**DOI:** 10.3389/fpsyg.2018.00521

**Published:** 2018-04-12

**Authors:** Giovanna Mioni, Franca Stablum, Simon Grondin, Gianmarco Altoé, Dan Zakay

**Affiliations:** ^1^Department of General Psychology, University of Padova, Padova, Italy; ^2^School of Psychology, Laval University, Quebec City, QC, Canada; ^3^Department of Developmental Psychology and Socialisation, University of Padova, Padova, Italy; ^4^New School of Psychology IDC, Herzliya, Israel

**Keywords:** time perception, time reproduction, children, symbolic meaning, speed

## Abstract

The present study investigated how the symbolic meaning of speed affects time perception in children and adults. We employed a time reproduction task in which participants were asked to reproduce temporal intervals previously presented. In Experiment 1, 45 primary school children and 22 university students performed a time reproduction task with cars (meaning of fastness) and trucks (meaning of slowness) presented for 11 and 21 s in static and moving conditions. Results showed that young children under-reproduced the duration more than the older children and adults, especially when the stimulus presented was a car. Moreover, participants under-reproduced moving stimuli compared to static one. In Experiment 2, we tested 289 participants who were divided into nine different age groups according to their school class: five from primary school, three from Junior High, and one from the university. Participants performed a time reproduction task with a motorbike (meaning of fastness) or a bicycle (meaning of slowness) under static and moving conditions for 11, 21, and 36 s. The results confirmed the effects of symbolic meaning of speed on children’s time perception and showed that vehicles that evoked the idea of fastness were under-reproduced compared to stimuli evoking the idea of slowness.

## Introduction

Time is always embedded in and inseparable from any human experience; despite children and adults are able to accurately perceive and process time, many different factors can alter and influence the subjective temporal experience. In fact, the perceived duration of an interval is prone to several contextual effects. Among these, there is one linked to the memory process involved in temporal processing. More specifically, one critical contextual factor is part of semantic memory, namely the meaning assigned to a certain situation (Droit-Volet et al., 2013; Palumbo et al., 2014; Zhang et al., 2014; Mioni et al., 2015).

According to Gibbon et al. (1984), temporal processing is based on a three-stage process (clock, memory, and decision), referred to as the scalar timing model. At the clock stage, the pacemaker emits pulses that pass through a switch and go into an accumulator. The memory stage is the storing system that collects pulses to be subsequently compared with the content in reference memory (decision stage). The estimated duration (subjective time) relates to the number of pulses accumulated during the stimulus to be timed: the more pulses accumulated, the more likely the stimulus duration will be judged as long. When attentional resources are allocated to time, more pulses are transmitted to the accumulator, producing a more accurate representation of time (Attentional Gate Model; Zakay and Block, 1996).

### Symbolic Meaning of Speed

Every stimulus we perceive is subjected to a semantic analysis (McKoon and Ratcliff, 1989). For example, if we see a tortoise or a hare the concept of slow and fast speed can be activated. The question addressed in the present study is to what extent semantic processing influence the temporal processing of the stimuli. In particular, we have focused on the effect of symbolic meaning of speed (real or implied movement) on time perception, and we investigated whether this might influences time perception. Actually, the concept of speed has recently been shown to affect duration judgments of young adults (Mioni et al., 2015).

Previous studies have shown that the time is not constant, but varies depending on the meaning assigned to a certain situation (Droit-Volet et al., 2013; Zhang et al., 2014; Mioni et al., 2015). Semantic meaning may play a crucial role on time perception. Palumbo et al. (2014) designed a study to understand what aspect of visual complexity has an impact on the perceived duration of a visual stimulus briefly presented. Palumbo et al. (2014) found that the semantic content of the image is crucial in determining duration, not the spatial/structural aspects of the stimulus.

Previous studies have shown the effect of implied speed of an individual’s actions on estimation of event duration (Burt and Pöpple, 1996; Burt, 1999). The faster the actions in an event are perceived to be, the shorter the estimated event duration. This result could be interpreted as the fruit of a reconstructive process partly based on inferences about the relationship between action speed and event duration. More recently, Nather and Bueno (2008) showed the effect of movement on time perception by manipulating implied motion in static images. The reproduced duration changed as a function of the amount of implied movement suggested by the static pictures. Over-reproduction was greater for postures involving greater movements than for postures involving fewer movements. Sgouramani and Vatakis (2014) also used a time reproduction task to study the effect of naturalistic dynamic dance stimuli (a dancer performing ballet steps in fast and slow versions) on time perception. Overall, they observed that the fast versions of the dance stimuli were under-estimated more than the slow versions. Finally, Zhang et al. (2014) compared 5 fast-speed (i.e., rapid) and 5 slow-speed (i.e., slow) words to investigate the effect of implicit information on time perception. Results indicated that fast-speed words are judged to be longer than the duration of the slow-speed words.

Together, these studies showed that the implicit meaning of speed indicated by words or images could change the subjective perception of duration. The results support also the important role of embodiment in time perception, which is possibly due either to self-referential processing or to an increased efficiency in information processing. Indeed, the embodied cognition (“being there”) are general models of information processing (see also Chambon et al., 2008). When incorporated with predictions from embodied cognition models, temporal models posit that temporal judgments will be biased by the presentation of stimuli included into the category “fast vehicles.” In particular, if perceivers reproduce the sensory states of “being there” driving a fast vehicle they should also recall the idea of going fast and reach faster a destination compared to driving a slow vehicle.

### A Developmental Perspective

An important transition period for processing information about time occurs between 6 and 8 years old when children’s cognitive resources are more developed and they have learned how to count (Clément and Droit-Volet, 2006; Droit-Volet et al., 2015; Droit-Volet, 2016). Most children under 7-years old have not learned to use chronometric units (seconds, minutes, and hours) and this learning takes place at different rhythms (Pouthas, 1993). In fact, 6-year old children are more erratic and variable than 8-year old children when tested with time reproduction and verbal estimation tasks (Droit-Volet and Wearden, 2001; Espinosa-Fernández et al., 2004; Droit-Volet, 2016). Consequently, the temporal performance of children shows greater variability, both inter-group as well as intra-group, when compared with that shown by adults (Pouthas, 1993). The acquisition of explicit time knowledge, at around 7 (Droit-Volet et al., 2001; Espinosa-Fernández et al., 2004; Zélanti and Droit-Volet, 2011), helps children to develop the awareness of the importance of time, and to use the temporal counting strategies properly.

No previous studies have investigated if the effects of the symbolic meaning of speed on time perception is different in children (at different ages) and in younger adults. The symbolic representation of speed might affect time perception differently in children and adults as temporal processing, as well as the effect of context, evolves with age and is more pronounced in children than in adults (Droit-Volet, 2016). The perception-action skills undergo a prolonged period of development, particularly when the task involves moving oneself in relation to other fast-moving objects in the environment (Plumert et al., 2007). There is evidence that even 14-year-olds are less skilled than adults, suggesting that extensive experience with moving oneself in relation to other fast-moving objects is critical for fine-tuning the perception of dynamic affordances (Plumert and Kearney, 2014).

### The Present Study

In the present investigation, we have focused, in a developmental perspective, on the effect of speed (real or implied movement) on time perception. Considering the tight relationship between speed, time and space (Piaget, 1979), showing pictures of vehicles involves the activation of the knowledge that more or less speed means more or less distance in less or more time. If the distance is maintained constant, we can reach a destination in a shorter time if we go faster (Matsuda, 1974). If the symbolic meaning of the stimulus acts on the pacemaker, when a stimulus representing the meaning of slowness is displayed, the rate of pulses’ emission should decrease, but when the presented stimulus is representative of fastness, the rate should increase. Within the theoretical framework of embodied cognition, participants might embody the slow speed and therefore have slowed down the speed of their internal clock (Chambon et al., 2008). However, if the meaning of the stimulus presented acts at the memory stage (semantic memory) we should observe a different pattern of performances. In such a case, if the representation stored in memory about objects’ speed acts on temporal estimation, showing a stimulus recalling the symbolic meaning of fast speed should lead to shorter perceived duration than showing a stimulus recalling the symbolic meaning of slow speed. Inferential processes may create individual differences in event duration estimates. There is, in fact, ample research demonstrating that specific characteristics of the stimulus can influence reconstructive outcomes. This inferential process can be explained by the space-time interaction and this interpretation is consistent with the results reported by Loftus and Palmer (1974) and Burt and Pöpple (1996).

Considering the use of stimuli recalling the meaning of speed-movement, we decided to control for the possible effect of real movement by presenting static and moving stimuli. Two possible outcomes are expected depending on the effect of movement at the pacemaker or attentional level. If the effect of motion on time perception is due to variation at the level of the pacemaker, we should expect an over-estimation of the duration of an interval containing moving stimuli when compared to static stimuli. One theoretical explanation for this outcome refers to arousal. Nather et al. (2011) showed that the perceived duration changed as a function of the amount of movement suggested by the static pictures representing the Degas dancers (Nather et al., 2011; Nather and Bueno, 2012; see also Sgouramani and Vatakis, 2014). On the other hand, if the effect of motion on time perception is due to the reduction of attentional resources dedicated to time, we should expect an underestimation in the moving stimuli condition compared to the static stimuli condition. As mentioned, the Attentional Gate Model (Zakay and Block, 1996) predicts that temporal performance depends on the amount of attentional resources dedicated to time. With less attention dedicated to time, less temporal information can enter in the accumulator, which results in an underestimation of time (Zakay and Block, 1996; Block et al., 2010). Some studies have demonstrated that motion easily captures attention (Hillstrom and Yantis, 1994; Franconeri and Simons, 2003); consequently, it is reasonable to assume that less attentional resources are dedicated to time perception when motion is observed, which lead to an under-reproduction of the presented duration.

Lastly, we also predicted developmental differences in temporal processing due to the developmental differences in the cognitive functions involved in temporal processing (i.e., attention, working memory; Espinosa-Fernández et al., 2004; Clément and Droit-Volet, 2006; Zélanti and Droit-Volet, 2011; Droit-Volet et al., 2015). In particular, the effect of symbolic meaning of speed would be more pronounced in younger children based on the observation that their temporal processing is highly sensitive to contextual effects (Droit-Volet, 2016). Older children, as well as adults, should have learnt strategies to compensate for the effect of the content on their subjective experience (Block et al., 1999).

## Experiment 1

### Method

#### Participants

Children were tested in their elementary school (Scuola Primaria di Primo Grado “Educandato San Benedetto”, Montagnana, Padova, Italy) while university students were tested at the Department of General Psychology, University of Padova, Italy.

Sixty-seven participants were included in the present study: 13 6-year old children (*M* = 6.38; *SD* = 0.51; female = 8), 16 7-year old children (*M* = 7.31; *SD* = 0.48; female = 10), 16 8-year old children (*M* = 8.3; *SD* = 0.48; female = 7), and 22 University students (*M* = 22.95; *SD* = 1.56; female = 17). None of the children tested have developmental disorders.

The study received the approval from the ethic committee of Department of General Psychology (Padova, Italy) and by the head of the school. Parents of the children received an information letter that described the experimental procedure and gave their written consent to include their children in the study. Children with developmental disorders were excluded from the analyses. An oral informed consent was also obtained from the University student participants.

#### Time Reproduction Task

**FIGURE 1:**
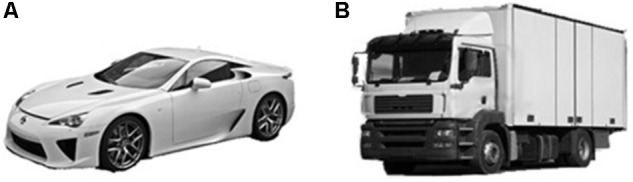
Vehicles used in the time reproduction task of Experiment 1: **(A)** car and **(B)** truck.

E-Prime^®^2.0 (Schneider et al., 2002) was used to program and run the experiment. The time reproduction task was presented on a 15-inch computer monitor and participants were tested individually in a quiet room, seated approximately 60 cm away from the computer screen. Participants were instructed to reproduce the duration of a previously seen stimulus. The stimuli were short movies that represented a sport car and a truck (see **Figure 1**) presented for either 11 or 21 s in random order. The stimuli were presented in black and white on a gray background. The size was 4 cm × 2 cm for the car and 4 cm × 3 cm for the truck. Stimuli either moved from the top right corner of the computer screen to the lower left corner of the computer screen (moving condition) or were presented at the center of the computer screen (static condition). The computer screen (diagonal) was 39 cm and this represented the hypothetical street were the vehicles moved. The speed of the moving stimuli was 3.54 cm/s when they were presented for 11 s, and 1.86 cm/s when presented for 21 s.

After the presentation of the stimulus, a question mark appeared indicating the beginning of the reproduction phase. Participants were taught to press the spacebar for the same amount of time they believed that the car or the truck was on the screen. During the reproduction phase, a circle appeared on the screen for the same amount of time the participants kept pressed the space bar. Participants completed 32 reproductions (32 trials), i.e., 4 repetitions of each conditions (2 *Standard durations*, 2 *Vehicles*, 2 *Movements*). A practice phase was included before the testing phase in which participants were instructed to reproduce 4 stimuli (2 in the static and 2 in the moving condition) that lasted 5 s; the stimuli in the practice phase indicated common objects (e.g., glass, hat, guitar, and shoe). At the end of the practice phase, participants were asked to repeat the instructions to confirm that they understood the task. All participants (children and adults) understood the instruction and were able to perform the task; no feedback was provided.

#### Statistical Analyses

We used a mixed-effects model approach (Pinheiro and Bates, 2000) considering its efficacy in dealing with complex data (Baayen et al., 2008; Di Giorgio et al., 2012). This approach allowed us to simultaneously consider all the factors that potentially could contribute to the understanding of the structure of the data. These factors include not only the standard fixed-effects factors controlled by the experimenter, but also the random effect factors (i.e., participants) characterized by the fact that their levels are randomly drawn from a population. The dependent variable was the mean of the 4 presentations of each condition and was analyzed in term of relative error (Ratio; Mioni et al., 2014). The Ratio reflects the direction of the timing error and is measured as the participant’s reproduced duration (Rd) divided by the target duration (Td) (Ratio = Rd/Td). Thus, scores of 1 equal perfect reproductions, while scores above 1.00 reflect over-reproductions, and scores below 1.00 reflect under-reproductions.

First, we estimate a baseline linear mixed-effects model with Ratio as dependent variable, *Age* g*roup* (6-, 7-, 8-year, and adults) as between-subject fixed effect, *Standard duration* (11 and 21 s), *Vehicle* (car and truck), and *Movement* (moving and static) as within-subject fixed effects, and Subjects (*n* = 67) as random effect. The four-way interaction and all two-way and three-way interactions between fixed effects were also included in the model.

Starting from the baseline theoretical model, we used a model selection approach based on Akaike Information Criterion (*AIC*) to find the most plausible model based on the observed data. To increase interpretability of results, an evidence ratio comparing the *AIC* of the best fitting model and the *AIC* of the baseline model was also calculated (Akaike, 1998; Wagenmakers and Farrell, 2004; McElreath, 2016).

To assess the statistical significance (at 0.05 level) of the effects included in the best fitting model, an analysis of deviance was performed. Significant interaction effects were graphically displayed. Furthermore, these effects were investigated in terms of simple effects (De Rosario-Martinez, 2015) via multiple contrasts adjusted with the Benjamini and Hochberg (1995) procedure.

Analyses were performed using *R* software (R Core Team, 2016). In particular: the package *lme4* (Bates et al., 2014) was used to estimate mixed-effects model via maximum likelihood, the package *car* (Fox et al., 2016) was used to perform analysis of deviance and the package *phia* (De Rosario-Martinez, 2015) was used to explore the interaction effects.

### Results

Observed means as a function of *Age group*, *Standard duration*, *Vehicle*, and *Movement* are reported in **Table 1**.

**Table 1:** Observed means and standard deviation of ratio in 6-, 7-, 8-years old children and adults as a function of duration (11 and 21 s), movement (static and moving), and vehicle (car and truck).

	11 s				21 s			
	Static		Moving		Static		Moving	
	Car	Truck	Car	Truck	Car	Truck	Car	Truck
	M (*SD*)	M (*SD*)	M (*SD*)	M (*SD*)	M (*SD*)	M (*SD*)	M (*SD*)	M (*SD*)
6-Years	0.45 (0.41)	0.49 (0.52)	0.48 (0.30)	0.53 (0.40)	0.25 (0.28)	0.43 (0.47)	0.22 (0.15)	0.38 (0.35)
7-Years	0.68 (0.32)	0.74 (0.30)	0.65 (0.34)	0.57 (0.30)	0.44 (0.29)	0.55 (0.21)	0.40 (0.29)	0.47 (0.21)
8-Years	0.83 (0.38)	0.75 (0.43)	0.74 (0.28)	0.63 (0.29)	0.55 (0.35)	0.69 (0.36)	0.48 (0.36)	0.65 (0.30)
Adults	0.98 (0.09)	0.99 (0.07)	0.99 (0.12)	0.99 (0.07)	0.96 (0.07)	1.00 (0.06)	0.99 (0.10)	0.96 (0.07)

**FIGURE 2:**
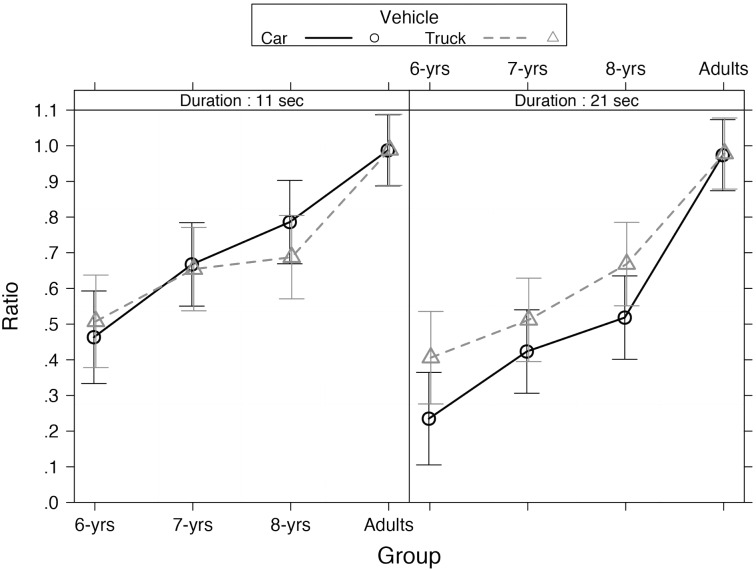
Estimated means of ratio by vehicle (car or truck) as a function of duration and age group in Experiment 1. Error bars represent 95% confidence intervals.

The best fitting model (AIC = -88.5) is presented in Appendix, Table A. The associated evidence Ratio showed that this model was 9775 times more likely to have generated the observed data than the baseline theoretical model (AIC = -70.1). The model included a significant main effect of *Movement* (χ^2^[1] = 5.80, *p* = 0.016), indicating that participants reproduced shorter duration in the moving condition compared to the static condition (static = 0.71, *SD* = 0.37; moving = 0.67, *SD* = 0.35). No interactions with movement were observed (all *p* > 0.05). Moreover, a three-way interaction between *Age group*, *Vehicle*, and *Standard duration* (χ^2^[3] = 8.23, *p* = 0.041), was also found (**Figure 2**). These effects were further investigated in terms of simple effects (De Rosario-Martinez, 2015) via multiple contrasts adjusted with the Benjamini and Hochberg (1995) procedure. Analysis across *Age group* showed that younger children reproduced shorter durations compared to older children and adults (all *p* < 0.001). Analysis across *Standard duration* showed shorter reproduced duration in 6-year old children (χ^2^[1] = 19.82, *p* < 0.001), 7-year old children (χ^2^[1] = 27.89, *p* < 0.001), and 8-year old children (χ^2^[1] = 33.56, *p* < 0.001) when the duration was 21 s compared to 11 s and the vehicle was a car. Shorter reproduced durations were observed in 7-year old children (χ^2^[1] = 9.48, *p* < 0.001) when the duration was 21 s compared to 11 s and the vehicle was a truck. Adults equally reproduced all durations independently of vehicle presented (all *p* > 0.05). Analysis across *Vehicle* showed shorter reproduced duration at 21 s in 6-year old children (χ^2^[1] = 11.13, *p* < 0.001) and 8-year old children (χ^2^[1] = 10.54, *p* < 0.001) when the vehicle was a car compared to truck.

### Discussion

The results showed that children generally under-reproduced temporal intervals, with younger children under-reproducing more than older children and adults. At 11 s, 6- and 7-year old children equally reproduced temporal intervals, but children who were 8-years old had performances closer to that of adults indicating better temporal ability than younger children. No effect of duration was observed in adults. These results are consistent with previous studies that found improvement in time judgments throughout childhood (Block et al., 1999; Chelonis et al., 2004; Droit-Volet, 2016).

More germane to the present study are the results that showed the effects of symbolic meaning of speed on time reproduction; children were influenced by the type of stimulus presented. In particular, we observed a larger under-reproduction when the stimulus was the car (meaning of fast speed) rather than the truck (meaning of slow speed) at 21 s. The results support that the symbolic meaning of speed influenced subjective perception of time. However, it is possible that the effects were related to the size of the stimuli (in real world). Xuan et al. (2007) demonstrated that large stimuli are perceived to last longer than smaller stimuli and concluded that magnitudes in temporal and non-temporal dimensions were not independent. This might explain the over-reproduction of the truck compared to the car in our study. To overcome this possible limitation, we conducted a second experiment with new stimuli: a motorbike (meaning of fast speed) and a bicycle (meaning of slow speed). The two new stimuli are closer in size in the real world and were presented at the same size during the experimental procedure.

## Experiment 2

Experiment 2 was conducted to replicate and extend the findings of Experiment 1 with different stimuli, a longer temporal interval, and more ages. We decided to include a longer temporal interval (36 s) because the symbolic meaning of speed effects observed in Study 1 were present only at 21 s and we reasoned that the effect may be greater at longer temporal intervals. Moreover, we decided to test older children to have a complete overview on the effects of symbolic meaning of speed and movement on time perception.

### Method

#### Participants

Children were tested in their schools (Scuola Primaria di Primo Grado di Via Moro, Campodarsego, Padova, Italy; Scuola Primaria di Primo Grado “Ruzante” Vigonza, Padova, Italy and Scuola Secondaria di Primo Grado “Don Lorenzo Milani”, Vigonza, Padova, Italy).

Two-hundred and eighty-nine participants were included in the present study: 160 participants were in elementary school: 29 6-year old children (*M* = 6.37; *SD* = 0.49; female = 20), 32 7-year old children (*M* = 7.25; *SD* = 0.44; female = 25), 33 8-year old children (*M* = 8.09; *SD* = 0.67; female = 27), 35 9-year old children (*M* = 9.00; *SD* = 0.64; female = 25), and 31 10-year old children (*M* = 10.10; *SD* = 0.79; female = 20). One hundred and three participants were in Junior High: 40 11-year old children (*M* = 11.08; *SD* = 0.86; female = 20), 33 12-year old children (*M* = 12.06; *SD* = 0.83; female = 19), and 30 13-year old children (*M* = 13.17; *SD* = 0.70; female = 22). Finally, 26 university students (*M* = 22.67; *SD* = 1.89; female = 14) were included in the study. Children with developmental disorders were excluded from the analyses. The ethic approval and the procedures for informed consent were the same as for Experiment 1.

#### Time Reproduction Task

The experimental procedures and materials were similar to those of Experiment 1 and the changes were as follows. First, the stimuli presented were movies that showed a motorbike and a bicycle (example of the stimuli used can be found in Mioni et al., 2015). The size was 4 cm × 2 cm for both stimuli. Three durations (11, 21, and 36 s) were employed and presented in random order. The speed of the moving stimuli was 3.54 cm/s when they were presented for 11 s, 1.86 cm/s when presented for 21 s, and 1.08 cm/s when presented for 36 s. Participants completed 48 reproductions (48 trials), which included 4 repetitions of each of the 12 conditions (3 *Standard durations*, 2 *Vehicles*, 2 *Movements*). The practice phase was identical to that of Experiment 1.

#### Statistical Analyses

Data were analyzed as in Experiment 1, with *Age group* (6-, 7-, 8-, 9-, 10-, 11-, 12-, 13-year old children, and adults) as between-subject fixed effect, *Standard duration* (11, 21, 36 s), *Vehicle* (motorbike and bicycle) and *Movement* (moving and static) as within-subject fixed effects, and *Subjects* (*n* = 289) as the random effect.

### Results

Observed means as a function of *Age group*, *Standard duration*, *Vehicle*, and *Movement* are reported in **Table 2**.

**Table 2:** Observed means and standard deviation in elementary school (6-, 7-, 8-, 9-, and 10-years old children), junior high (11-, 12-, and 13-years old children) and adults as a function of duration (11, 21, and 36 s), movement (static and moving), and vehicle (motorbike and bicycle).

	11 s				21 s				36 s			
	Static		Moving		Static		Moving		Static		Moving	
	Motorbike	Bicycle	Motorbike	Bicycle	Motorbike	Bicycle	Motorbike	Bicycle	Motorbike	Bicycle	Motorbike	Bicycle
6-Years	0.84 (0.45)	0.72 (0.36)	0.71 (0.39)	0.71 (0.32)	0.47 (0.25)	0.61 (0.30)	0.47 (0.23)	0.47 (0.24)	0.35 (0.24)	0.48 (0.25)	0.32 (0.17)	0.38 (0.24)
7-Years	0.87 (0.34)	0.90 (0.48)	0.74 (0.20)	0.87 (0.36)	0.72 (0.31)	0.80 (0.31)	0.64 (0.29)	0.72 (0.35)	0.65 (0.34)	0.73 (0.29)	0.54 (0.27)	0.57 (0.29)
8-Years	0.88 (0.24)	0.87 (0.23)	0.77 (0.23)	0.81 (0.29)	0.80 (0.19)	0.90 (0.24)	9.69 (0.23)	0.76 (0.23)	0.71 (0.30)	0.81 (0.23)	0.74 (.23)	0.74 (0.26)
9-Years	0.89 (0.13)	0.95 (0.15)	0.89 (0.20)	0.90 (0.15)	0.90 (0.13)	0.92 (0.12)	0.87 (0.20)	0.84 (0.18)	0.82 (0.18)	0.89 (0.16)	0.74 (0.24)	0.82 (0.15)
10-Years	0.89 (0.16)	0.89 (0.16)	0.81 (0.17)	0.87 (0.19)	0.90 (0.14)	0.90 (0.14)	0.84 (0.17)	0.84 (0.16)	0.80 (0.13)	0.88 (0.13)	0.77 (0.16)	0.80 (0.15)
11-Years	0.93 (0.13)	0.91 (0.15)	0.83 (0.14)	0.87 (0.16)	0.95 (0.21)	0.90 (0.12)	0.89 (0.19)	0.89 (0.16)	0.82 (0.19)	0.91 (0.14)	0.84 (0.20)	0.82 (0.16)
12-Years	0.91 (0.14)	0.91 (0.14)	0.84 (0.12)	0.86 (0.16)	0.87 (0.17)	0.91 (0.15)	0.84 (0.17)	0.87 (0.17)	0.84 (0.14)	0.91 (0.13)	0.83 (0.15)	0.84 (0.15)
13-Years	0.91 (0.14)	0.90 (0.10)	0.88 (0.16)	0.86 (0.18)	0.93 (0.15)	0.88 (0.20)	0.87 (0.16)	0.90 (0.15)	0.85 (0.19)	0,86 (0.16)	0.81 (0.25)	0.83 (0.18)
Adults	0.06 (0.11)	1.06 (0.14)	1.06 (0.18)	1.04 (0.13)	1,04 (0.14)	1.02 (0.10)	0.98 (0.12)	0.98 (0.10)	0.98 (0.08)	0.98 (0.08)	0.95 (0.12)	0.96 (0.12)

The best fitting model (AIC = -92.9) is presented in Appendix, Table B. The associated evidence ratio showed that this model was more than 10000 times more likely to have generated the observed data than the baseline theoretical model (AIC = -86.9).

**FIGURE 3:**
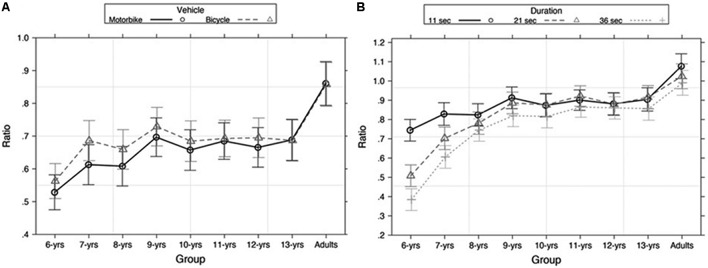
Estimated means of ratio for **(A)** vehicle (motorbike and bicycle) as a function of age group; estimated means ratio for duration (11, 21, and 36 s) as a function of age group **(B)** in Experiment 2. Error bars represent 95% confidence intervals.

**FIGURE 4:**
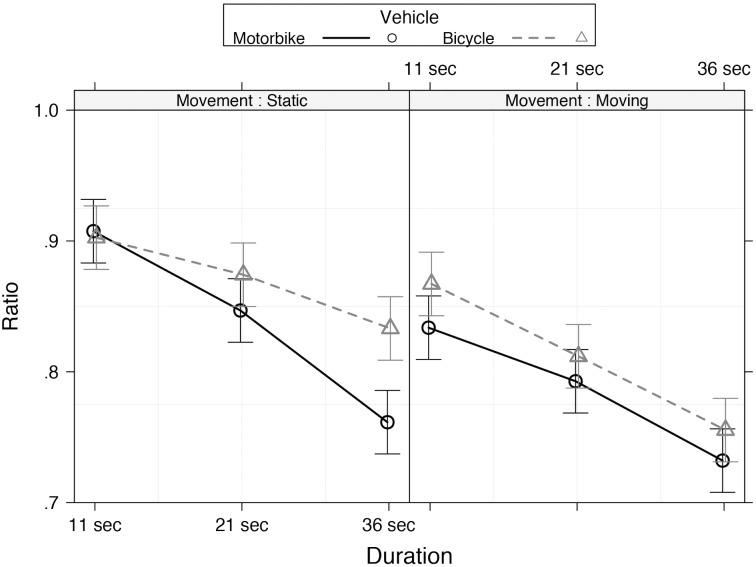
Estimated means of ratio for vehicle (motorbike and bicycle) as a function of duration (11, 21, and 36 s) and movement (moving and static) in Experiment 2. Error bars represent 95% confidence intervals.

The model that best explained our data included two two-way interactions: (1) *Age group* and *Vehicle* (χ^2^[8] = 16.69, *p* = 0.034); (2) *Age group* and *Standard duration* (χ^2^[16] = 238.56, *p* < 0.001) (**Figures 3A,B**). A three-way interaction between *Standard duration*, *Vehicle*, and *Movement* (χ^2^[2] = 9.94, *p* < 0.001) (**Figure 4**) was also observed. These effects were further investigated as in Experiment 1.

(1) Shorter reproduced durations were observed in children between 6- and 8-years old when stimuli were motorbike (all *p* < 0.001). All other participants equally reproduced temporal intervals when the stimuli were motorbike or bicycle (all *p* ≥ 0.05) (**Figure 3A**). (2) Younger children (between 6 and 8 years old) under-reproduced time more than older children and adults; moreover, all participants produced shorter durations as the duration increased (all *p* < 0.001) (**Figure 3B**).

Considering the three-way interaction, analysis across *Standard duration* showed shorter reproduced duration in all participants as the duration increased (all *p* < 0.001). Analysis across *Vehicle* showed shorter reproduced duration at 36 s in the static condition when the stimulus presented was a motorbike compared to a bicycle (χ^2^[1] = 26.78, *p* < 0.001). Finally, analysis across *Movement* showed shorter reproduced duration in the moving compared to static condition in all vehicles (all *p* < 0.05).

### Discussion

Results of Experiment 2 confirmed and extended the findings of Experiment 1. Younger participants under-reproduced temporal intervals, and the under-reproductions were larger with the increased duration of the stimulus. Regarding the effect of symbolic meaning, the results showed that the presentation of a vehicle that insinuates the idea of fast and slow speed can affect time perception. In particular, the meaning of fast speed (motorbike) led to larger under-reproductions than the meaning of slow speed (bicycle). The effect of the symbolic meaning of speed on time perception can be observed even when controlling for differences in the size of objects. Again, the effect of symbolic meaning of speed on time perception is only evident in younger children until 8 to 9-years old and disappeared in older children and adults.

Regarding the effect of movement on time perception, results substantiated and extended the findings of Experiment 1. Moving stimuli led to larger under-reproductions than using static stimuli, and the movement condition interacted significantly with duration. When the stimuli were static bicycles, no differences between durations were observed, whereas when the stimuli were static motorbikes, participants under-reproduced longer stimulus (21 and 36 s).

## General Discussion

### Symbolic Meaning

When we judge the passage of time, our temporal experience can be biased in a number of ways. For example, the presentation of a face on a computer screen is perceived to last longer when the it expresses anger than when it expresses a neutral emotion (Droit-Volet et al., 2013). Here, for the first time we showed the effect of symbolic meaning of speed on children’s time processing. Stimuli that recalled the semantic meaning of fast speed (car and motorbike) were more under-reproduced than the stimuli that evoked the symbolic meaning of slow speed (truck and bicycle). Importantly, we showed that this effect was independent of the real-world size of the objects (Experiment 2).

Our findings are in line with what was observed regarding the remembered duration of long events (reconstructive process; Burt and Pöpple, 1996; Burt, 1999) and with the embodied cognition approach (Garbarini and Adenzato, 2004). For example, using the temporal bisection task, Chambon et al. (2008) reported a temporal under-estimation when the stimuli were faces of elderly individuals compared to that of faces of young individuals. They discussed their results within the theoretical framework of embodiment by suggesting that the participants embodied the slow movements of elderly people and that produced a consecutive effect on temporal judgment.

However, our effect of symbolic meaning was evident only in children (in particular from ages 6 to 8) and had disappeared in older participants. This might be explained by the fact that for children, time judgments are often context-dependent, that is, they depend on salient non-temporal information. Younger children may be more sensitive than are adolescents and adults, to the stimulus content. Probably because they have not yet learned how various factors influence duration experience and they have selective difficulties regarding inhibiting irrelevant information. Older children, adolescents, and adults may be compensating for the effect of the content on their subjective experience (Block et al., 1999).

The lack of effect of symbolic meaning on temporal performances in older children and adults might also be explained by the temporal task employed. Participants engaged in a time reproduction task with long temporal intervals are prone to use strategies (i.e., counting) to perform the task (Clément and Droit-Volet, 2006). To prevent counting strategies, secondary concurrent non-temporal tasks (Mioni et al., 2014) or very brief intervals are employed (Grondin et al., 1999, 2004). The focus of the present investigation was on the effect of speed (real or implied movement) on time perception in a developmental perspective, and therefore we decided not to include secondary tasks or use of very short intervals. Therefore, older children and adults might easily have used additional cognitive strategies to perform the tasks and this could have covered the effect of symbolic meaning on their subjective time perception.

These conclusions are supported by a recent study conducted with adults using a time bisection task with temporal intervals ranging from 400 to 1600 ms (Mioni et al., 2015). Participants first learned the two standard intervals (standard short = 400 ms and standard long = 1600 ms) and then were instructed to judge the new temporal intervals presented as being closer to the standard short or to the standard long. The study showed that the effect of the symbolic meaning of speed (fast/slow) on time perception is also evident in adults. These results confirmed the effect of symbolic meaning on time perception and highlighted the importance of the different temporal tasks (see Gil and Droit-Volet, 2011; Mioni et al., 2014).

### Moving vs. Static Stimuli

Our results showed that participants generally under-reproduced moving stimuli in relation to static stimuli suggesting that the effect of real movement on time perception could be explained by a variation at the attentional level rather than by an effect on the pacemaker. According to the Attentional Gate Model (Zakay and Block, 1996), when participants are engaged in a time reproduction task and attentional resources are subtracted from temporal processing, less pulses are accumulated, leading to under-reproduction of temporal intervals. Indeed, some studies have shown that motion stimuli capture more attention compared to static stimuli and this is evident in both adults (Hillstrom and Yantis, 1994; Franconeri and Simons, 2003; Lin et al., 2009) and children (Humphrey et al., 1986). Studies on the importance of motion have been influenced strongly by Gibson’s proposal that temporal transformations of the optic array can provide far richer information about the visual world than the projection of a single static image into retinae (Gibson, 1966; for a review, see Humphrey et al., 1986). If that is the case, we can contend that attention is more attracted by moving stimuli and, therefore, less attention is dedicated to time when the stimulus is moving. This leads to a larger under-reproduction in conditions with moving stimuli than in conditions with static stimuli. Therefore, presenting stimuli in a moving condition may capture more attention than presenting stimuli under a static condition, and this might have caused the observed under-reproduction (Zakay, 1992). Interestingly, in Experiment 2, the under-reproductions observed were larger for the longer temporal intervals (21 and 36 s). Additionally, Tayama and Aiba (1982) and Tayama et al. (1987) found that the slowest moving stimuli in a set were judged to be shorter in duration than stationary stimuli. These findings are consistent with our results (Experiment 2) which show greater under-reproduction for moving stimuli at 36 s (slower speed).

Given the prediction by the Attentional Gate Model and considering that, younger children do have lower attentional resources (Droit-Volet et al., 2015), we would have expected greater under-reproduction of moving stimuli in younger participants than in older children and adults. However, we did not observe a greater under-reproduction in younger children when the moving stimuli were presented and the effects of movement were equally present in both children and adults. The human visual system is known to have specialized motion-processing abilities; moreover, moving stimuli automatically attracts more attention in order to prioritize the processing of the information associated with motion. Thus, in both children and adults, attention is automatically attracted by moving stimuli, which might explain the similarity in under-reproduction in both children and adults when moving stimuli are presented (Brown, 1995; Keshavarz et al., 2010; Lewis and Neider, 2015).

### Age Effect

Finally, our results showed a developmental change in time processing. Younger children showed a greater difference from the objective time than older children and adults. Age-related improvement of temporal performances is a well-known phenomenon (Droit-Volet, 2016). By age 7, the acquisition of explicit time knowledge (Droit-Volet et al., 2001; Espinosa-Fernández et al., 2004; Zélanti and Droit-Volet, 2011) helps children both to develop awareness of the importance of time estimation, and to implement temporal strategies. Pouthas et al. (1990) showed that before the age of 10, most children do not spontaneously use explicit timing-related strategies. Younger children may have more limited attention resources than Junior High school children and adults. In addition, differences in memory processes, such as the rate of forgetting of information, might explained the differences between children and adults in temporal processing (Block et al., 1999).

In both experiments, we also observed that temporal improvement in children is not constant, but depends on the temporal intervals under investigation. In fact, a smaller discrepancy was observed when the temporal interval lasted 11 s, as compared to longer temporal intervals (21 and 36 s). Zélanti and Droit-Volet (2011) showed that 9-year old children achieved a level of time sensitivity close to that observed in adults for durations shorter than 2.5 s. However, when the durations were longer than 2.5 s their sensitivity to time remained low. These findings suggest that the development of children’s ability to discriminate time takes the form of an increased capacity to process shorter and longer durations accurately and thus resembles an increase in temporal span. This increased capacity might be related to the acquisition of temporal strategies and the improvements in cognitive processes related to time (Zélanti and Droit-Volet, 2011). Interestingly, in our study, children around the age of 9/10-years old performed similarly to Junior High school children when short temporal intervals were involved. Junior High school children reproduce time similar to adults throughout all temporal intervals.

### Limitations

A limitation of the present study is the low number of trials per condition (four repetitions). We decided to avoid overloading children, in particular because we used relatively long temporal intervals and children have limited attentional resources (Zélanti and Droit-Volet, 2011; Droit-Volet et al., 2015). Moreover, we followed previous studies (Meaux and Chelonis, 2003; Carelli et al., 2008) conducted with children that used time reproduction tasks.

## Conclusion

Taken together, our results confirmed lower sensitivity to time in children and showed an improvement in temporal performance associated with increased age. Regarding the effect of movement on time perception, we showed that, in both children and adults, moving stimuli were under-reproduced compared to static stimuli; these results were in accord with the Attentional Gate Model (Zakay and Block, 1996).

With regard to the effect of symbolic meaning on time perception, we found that stimuli that recalled the symbolic meaning of fast vehicles were under-reproduced more than stimuli that recalled the symbolic meaning of slow vehicles. Our results were interpreted in accord with an inferential/reconstructive process that occurred in memory and acted on temporal judgments and in line with the embodied theory.

Our results could have interesting implications for real-world situations. For example, in the situation in which a busy street has to be crossed, investigating the relationship between time perception, symbolic and real speed representations might have important implications for understanding children’s behavior (see Plumert and Kearney, 2014). This work does not resolve all issues on how the symbolic meaning of speed affects time perception, and leaves some open questions, especially regarding the mechanism by which the symbolic meaning of speed affects the pacemaker and time estimation. We believe that most of the differences between younger children and adults are due to variation at the attentional and memory functions involved in temporal processing, rather than due to variations at the level of the pacemaker (see also Droit-Volet et al., 2013). However, it should be emphasized that the paper reports new empirical observations, offers some explanations for them, and invite time researchers to further explore the topic, maybe testing this effect in auditory domain.

## Author Contributions

GA performed data analysis. FS, SG, and DZ provided ongoing contributions and feedback throughout the experimental process. They also provided additional revisions to the manuscript. GM drafted the manuscript and was involved in data collection. All the authors were involved in the conception of the work, approved the final version of the manuscript, and agreed to be accountable for all aspects of the work.

## Conflict of Interest Statement

The authors declare that the research was conducted in the absence of any commercial or financial relationships that could be construed as a potential conflict of interest.
