# Supplementary material for: Effect of the Symbolic Meaning of Speed on the Perceived Duration of Children and Adults
Source: Front Psychol. 2018 Apr 12;9:521. doi: 10.3389/fpsyg.2018.00521 (PMC5932387; doi:10.3389/fpsyg.2018.00521)
Supplement: Supplementary file 1 [file Table_1.DOCX]

**APPENDIX**

**TABLE A**

Results of the logistic mixed-effects model with Ratio as dependent variable for Experiment 1.

|  | **β** | **Standard errors** | **χ^2^(*df*)** |
| --- | --- | --- | --- |
| **Fixed Parts** |  |  |  |
| **Age group** |  |  | 73.15 (3)*** |
| 7-years | 0.24*** | 0.09 |  |
| 8-years | 0.36*** | 0.09 |  |
| Adults | 0.52*** | 0.09 |  |
| **Duration** |  |  | 53.03 (1)*** |
| 21 sec | -0.23*** | 0.05 |  |
| **Vehicle** |  |  | 5.40 (1)* |
| Truck | 0.04 | 0.05 |  |
| **Movement** |  |  | 5.80 (1)* |
| Moving | -0.00 | 0.04 |  |
| **Age group x Duration** |  |  | 22.10 (3)*** |
| 7-years × 21 sec | -0.02*** | 0.07 |  |
| 8-yeras × 21 sec | -0.04*** | 0.07 |  |
| Adults × 21 sec | 0.21*** | 0.06 |  |
| **Age group x Vehicle** |  |  | 5.41 (3) |
| 7-years × Truck | -0.06 | 0.07 |  |
| 8-yeras × Truck | -0.14 | 0.07 |  |
| Adults × Truck | -0.04 | 0.06 |  |
| **Age group x Movement** |  |  | 6.31 (3) |
| 7-years × Moving | -0.08 | 0.05 |  |
| 8-yeras × Moving | -0.08 | 0.05 |  |
| Adults × Moving | 0.00 | 0.04 |  |
| **Vehicle x Duration** |  |  | 11.69 (1)*** |
| Truck × 21 sec | 0.13 | 0.07 |  |
| **Age group x Vehicle x Duration** | |  | 8.23 (3)* |
| 7-years × Truck × 21 sec | -0.02* | 0.10 |  |
| 8-yeras × Truck × 21 sec | 0.12 | 0.10 |  |
| Adults × Truck × 21 sec | -0.12* | 0.09 |  |

Baseline category for Age group was “6-year”; Baseline category for Duration was “11 sec”; Baseline category for Vehicle was “Car” and Baseline category for Movement was “static”. Random effect was Participants. Number of observations = 536. Number of participants = 67. **p*<.05, ***p*<.01, ****p*<.001.
