# Supplementary material for: Effect of the Symbolic Meaning of Speed on the Perceived Duration of Children and Adults
Source: Front Psychol. 2018 Apr 12;9:521. doi: 10.3389/fpsyg.2018.00521 (PMC5932387; doi:10.3389/fpsyg.2018.00521)
Supplement: Supplementary file 2 [file Table_2.DOCX]

**APPENDIX**

**TABLE B**

Results of the logistic mixed-effects model with Ratio as dependent variable for Experiment 2

|  | **β** | **Standard errors** | **χ^2^(*df*)** |
| --- | --- | --- | --- |
| **Fixed Parts** |  |  |  |
| **Age group** |  |  | 200.88 (8)*** |
| 7-years | 0.08*** | 0.04 |  |
| 8-years | 0.08*** | 0.04 |  |
| 9-years | 0.17*** | 0.04 |  |
| 10-years | 0.13*** | 0.04 |  |
| 11-years | 0.16*** | 0.04 |  |
| 12-years | 0.14*** | 0.04 |  |
| 13-years | 0.16*** | 0.04 |  |
| Adults | 0.33*** | 0.04 |  |
| **Duration** |  |  | 243.86 (2)*** |
| 21 sec | -0.25*** | 0.02 |  |
| 36 sec | -0.40*** | 0.02 |  |
| **Vehicle** |  |  | 25.37 (1)*** |
| Bicycle | 0.00 | 0.02 |  |
| **Movement** |  |  | 97.27 (1)*** |
| Moving | -0.07*** | 0.01 |  |
| **Age group x Duration** |  |  | 238.56 (16)*** |
| 7-years × 21 sec | 0.11*** | 0.03 |  |
| 8-years × 21 sec | 0.19*** | 0.03 |  |
| 9-years × 21 sec | 0.21*** | 0.03 |  |
| 10-years × 21 sec | 0.24*** | 0.03 |  |
| 11-years × 21 sec | 0.26*** | 0.03 |  |
| 12-years × 21 sec | 0.23*** | 0.03 |  |
| 13-years × 21 sec | 0.25*** | 0.03 |  |
| Adults × 21 sec | 0.18*** | 0.03 |  |
| 7-years × 36 sec | 0.14*** | 0.03 |  |
| 8-years × 36 sec | 0.28*** | 0.03 |  |
| 9-years × 36 sec | 0.27*** | 0.03 |  |
| 10-years × 36 sec | 0.30*** | 0.03 |  |
| 11-years × 36 sec | 0.32*** | 0.03 |  |
| 12-years × 36 sec | 0.34*** | 0.03 |  |
| 13-years × 36 sec | 0.31*** | 0.03 |  |
| Adults × 36 sec | 0.27*** | 0.03 |  |
| **Age group x Vehicle** |  |  | 16.69 (8)* |
| 7-years × Bicycle | 0.04* | 0.02 |  |
| 8-years × Bicycle | 0.02* | 0.02 |  |
| 9-years × Bicycle | -0.00* | 0.02 |  |
| 10-years × Bicycle | -0.01* | 0.02 |  |
| 11-years × Bicycle | -0.03* | 0.02 |  |
| 12-years × Bicycle | -0.00* | 0.02 |  |
| 13-years × Bicycle | -0.04* | 0.02 |  |
| Adults × Bicycle | -0.04* | 0.03 |  |
| **Vehicle x Duration** |  |  | 6.251 (2)* |
| 21 sec × Bicycle | 0.03*** | 0.02 |  |
| 36 sec × Bicycle | 0.08*** | 0.02 |  |
| **Movement x Duration** |  |  | 0.13 (2) |
| Moving × 21 sec | 0.02 | 0.02 |  |
| Moving × 36 sec | 0.02 | 0.02 |  |
| **Vehicle x Movement** |  |  | 0.29 (1) |
| Bicycle × Moving | 0.04* | 0.02 |  |
| **Movement x Vehicle x Duration** |  |  | 9.94 (2)** |
| Moving × Bicycle × 21 sec | -0.05** | 0.03 |  |
| Moving × Bicycle × 36 sec | -0.09** | 0.03 |  |

Baseline category for Group was “6-year”; Baseline category for Duration was “11 sec”; Baseline category for Vehicle was “Motorbike” and Baseline category for Movement was “Static”. Random effectwas Participants. Number of observations = 3469. Number of participants = 289. **p*<.05, ***p*<.01, ****p*<.001.
